# Optimisation of photonic crystal coupling through waveguide design

**DOI:** 10.1007/s11082-016-0888-0

**Published:** 2017-01-06

**Authors:** R. J. E. Taylor, P. Ivanov, G. Li, D. T. D. Childs, R. A. Hogg

**Affiliations:** 1 Department of Electronic and Electrical Engineering, The University of Sheffield, Sheffield, S3 7HQ UK; 2 Department of Electrical Engineering and Information Systems, School of Engineering, The University of Tokyo, 7-3-Hongo, Bunkyo-ku, Tokyo, 113-8656 Japan; 3 School of Engineering, The University of Glasgow, Glasgow, G12 8LT UK

**Keywords:** Photonic crystal, Laser, Waveguide

## Abstract

This paper considers multiple structural designs for photonic crystal surface emitting lasers operating at key wavelengths. Initially a structure from Williams et al. is modelled, the structure is modified to include an additional GaAs waveguide layer (termed ballast layer) and to include an additional PC layer (termed double decker). These structures are modelled by a combination of coupling calculation and waveguide modelling and are compared to the original structure. We show that both of these schemes give an increase in coupling, but present fabrication challenges. Next, we model standard laser structures operating at key wavelengths (400 nm, 1.3 and 10 µm) where a photonic crystal is located above the active region and explore the effect of increasing thickness of photonic crystal. We find that increasing the thickness increases the coupling coefficient but not true for the full range of thicknesses considered. This study allows a more universal comparison of the use of all-semiconductor, or void containing PCSELs to be conducted and we find that the realisation of all semiconductor PCSELs covering a wide range of material and wavelengths are possible.

## Introduction

There has been considerable recent interest in photonic crystal surface emitting lasers (PCSELs; Hirose et al. 2014; Miyai et al. 2006; Imada et al. 1999; Ohnishi et al. 2004; Sakai et al. 2005). In the PCSEL, lasing occurs through Bragg diffraction and in-plane feedback (Kurosaka et al. 2008) brought about by the periodic variation in refractive index of the photonic crystal (PC). PCSELs have been shown to give single mode emission over a large area (Hirose et al. 2014; Miyai et al. 2006; Imada et al. 1999; Ohnishi et al. 2004; Sakai et al. 2005), high power (Hirose et al. 2014), low divergence (Imada et al. 1999; Ohnishi et al. 2004), control of beam shape and polarisation (Kurosaka et al. 2008; Noda et al. 2001) and beam steering (Kurosaka et al. 2010). Typically PCSELs contain voids and are realised through wafer fusion (Noda et al. 2001; Imada et al. 1999) or through epitaxial regrowth of voids (Hirose et al. 2014). PCSELs offer some advantages over Fabry Pérot (FP), distributed feedback (DFB) and vertical cavity surface emitting lasers. In particular, they promise high brightness (Hirose et al. 2014; Imada et al. 1999) and power scaling whilst maintaining circular shape of beam and low divergence angles. Many of these properties are desirable in a wide range of applications, for example low divergence beams are desirable for working towards lens-less laser modules.

**Fig. 1:**
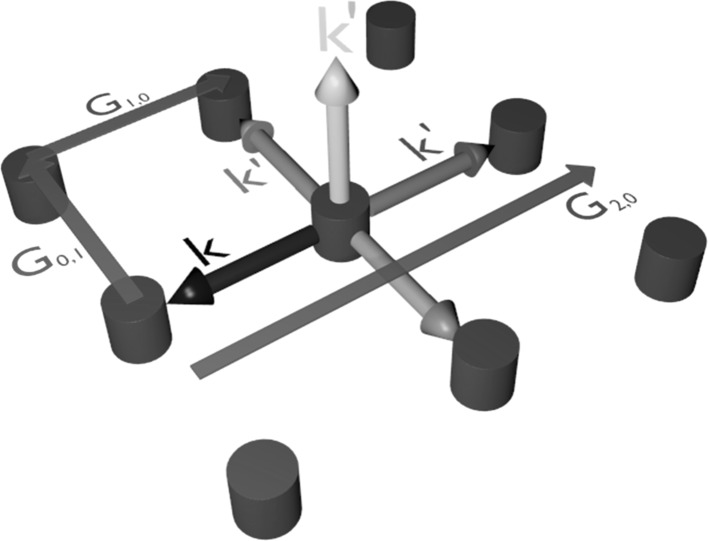
Schematic of PCSEL scattering directions

Within a photonic crystal laser, light traveling within in-plane will undergo multiple scattering events. Figure 1 shows a schematic of scattered light waves in a photonic crystal consisting of circular pillars on a square lattice, showing in plane and out of plane scattering. Light is scattered backward and forward, ±90° and out of plane. The four scattering directions are coupled and a 2D standing wave is formed. Light scattered out of plane becomes the lasing light emitted normal to the surface. The in-plane scattering of coupled light gives rise to lasing from the entire PCSEL surface and enables lasing from a large area. This leads to high power and low divergence (a requirement for high brightness).

Williams et al. (2012a, b) demonstrated the first all-semiconductor PCSEL based on epitaxial regrowth, which incorporates an all-semiconductor PC rather than a void containing PC. These devices were modelled as a 1D waveguide and were shown to have a higher mode overlap and coupling than similar void containing structures. This result was subsequently confirmed by Taylor et al. (2013).

**Fig. 2:**
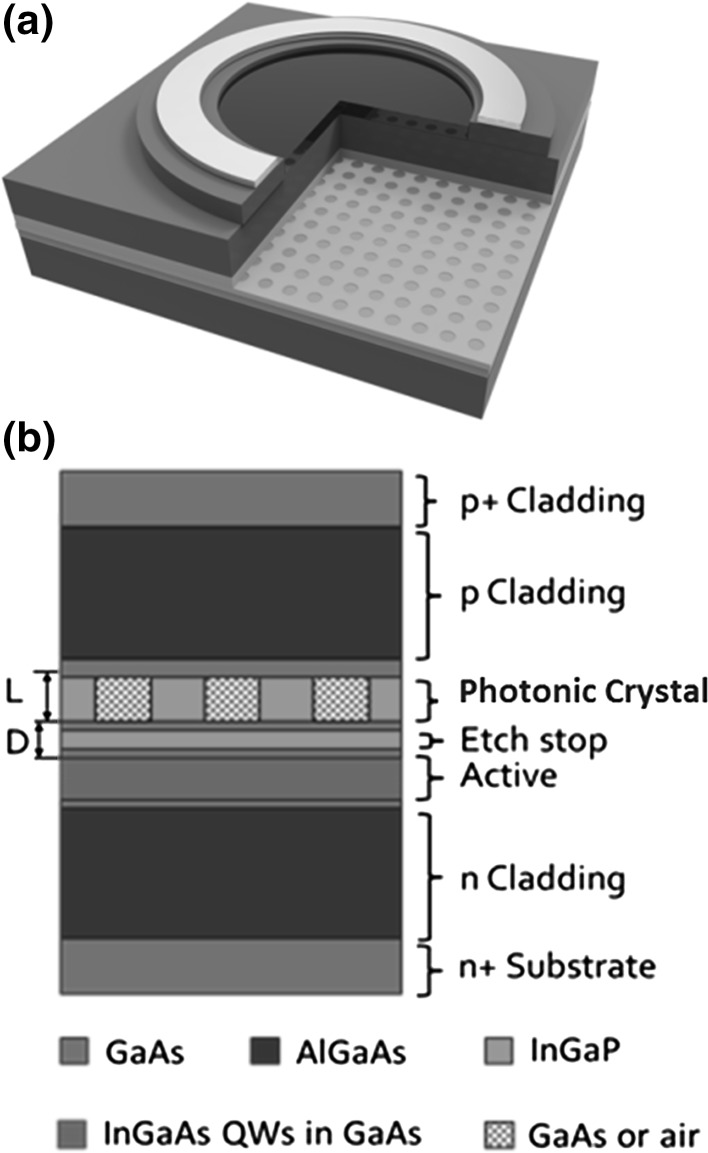
Schematic of PCSEL 3D cross section (**a**) and layer structure (**b**)

Figure 2a shows a 3D schematic of a PCSEL structure, whilst Fig. 2b shows the layer structure of the same device. PC layer thickness (L) and the separation between active elements and the PC layer (D) are highlighted. The structure considered is similar to Williams et al. (2012a, b) and consists of (from bottom to top) a n-type AlGaAs lower cladding layer, a three quantum well active layer consisting of three 8 nm InGaAs quantum wells separated by 20 nm GaAs layers, an etch stop layer, the photonic crystal region of 150 nm which is InGaP/GaAs for the all-semiconductor case and InGaP/air for the void containing case, a p-type AlGaAs cladding layer and finally a highly doped p-type GaAs layer. Table 1 shows the layer structure and refractive indices of the GaAs based PCSEL considered in this paper.

**Table 1:** Epitaxial layer structure of device considered

Material	Thickness (nm)	Refractive index
Al_0.4_Ga_0.6_As	3000	3.31
GaAs	T	3.521
GaAs	80	3.521
In_0.48_Ga_0.52_P/GaAs or In_0.48_Ga_0.52_P/void	L	n_av_
GaAs	20	3.521
In_0.48_Ga_0.52_P	40	3.143
GaAs	20	3.521
In_0.2_Ga_0.8_As	8	3.736
GaAs	20	3.521
In_0.2_Ga_0.8_As	8	3.736
GaAs	20	3.521
In_0.2_Ga_0.8_As	8	3.736
GaAs	30	3.521
Al_0.4_Ga_0.6_As	3000	3.31

Much of the work in modelling PCs has concentrated on the design of photonic crystal shape and lattice geometry (Sakai et al. 2006; Plihal and Maradudin 1991; Nielsen et al. 1999; Yokoyama and Noda 2003; Kurosaka et al. 2009), to date the effect of the waveguide design on PCSEL performance has not been comprehensively studied. This may in part be due to difficulties in engineering waveguides which contain the low average mode index associated with void containing PCSELs (Taylor et al. 2013).

Semiconductor lasers are ubiquitous. Short wavelengths are desirable for many applications including 405 nm for optical data storage (Mitsuhashi 1997), laser lighting, and biomedical applications (Brezinski 2006). 1.3 and 1.55 µm lasers are the workhorses of optical communications and optical gyroscopes (Lefevre 2014) because of the dispersion and absorption properties of silica fibre (Mitschke 2009). Infra-red (IR) quantum cascade lasers (QCLs) are attractive for a range of applications including security applications such as target illumination and counter measures, and for high sensitivity gas sensing (Werlea et al. 2001).

In this paper we consider an effective refractive index model for both all semiconductor (Williams et al. 2012a) and identical void containing designs. For this basic structure emitting at ≈980 nm, we initially consider the effect of PC radius on the confinement of the optical mode and overlap with the PC and active elements. In conventional laser design the mode overlap with the active region is considered as a key parameter, particularly in reducing threshold gain. For a PCSEL the coupling coefficient of the PC is a critical factor in achieving well separated (in wavelength) vertically emitting modes and in obtaining high power output per unit area. We find that the highest coupling to the grating occurs when the separation between the PC and active elements is small and in these regimes the overlap with the active is also maximised. As such this paper focuses on optimising the coupling coefficient of the structure alone.

The low effective refractive index of the void containing PC is highlighted as a key issue in PCSEL design. We go on to explore the use of an additional wave-guiding layer (ballast layer) above the PCSEL, and the use of a PC above and below the active element. Trends are assessed, and comparisons are made between all-semiconductor and void-containing PCSELs. General guidelines are deduced for PCSEL waveguide design. We show that the inclusion of a ballast layer increases the PC coupling for both all-semiconductor and void containing structures but that all-semiconductor PCSELs still have a higher coupling. The double decker structure is shown to increase the PC coupling and is the first structure in this study to give a higher coupling for void containing PCSELs than for all-semiconductor PCSELs, however we highlight the complexity in manufacturing such a device.

We go on to consider PCSEL designs for different material systems and wavelengths. The wavelengths considered are 405 nm (Kawashima et al. 2010) based in GaN, 1.3 µm (Imada et al. 1999) based on InP and 10 µm based on InP. We show that for standard edge-emitting designs for these key laser wavelength, PCSEL structures may be readily realised. We show how for all these designs, utilising a range of materials, and spanning a large spectral range, high PC coupling coefficients are obtained and thus it is possible for PCSELs to cover a full range of Laser applications.

## Definitions

**Fig. 3:**
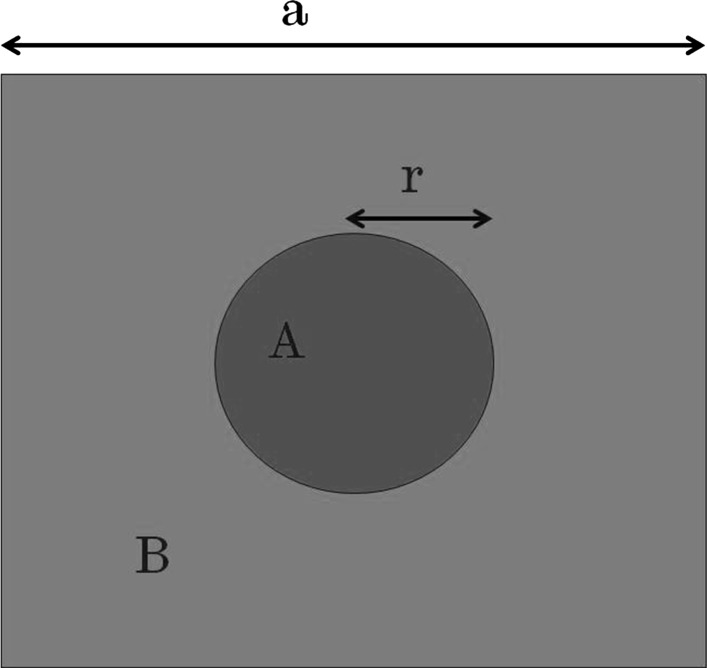
Schematic of a photonic crystal, indicating nomenclature used for atom and background material (*A* and *B* respectively), atom radius (*r*) and unit cell size (*a*)

Figure 3 shows a plan-view schematic of a photonic crystal where, key parameters are defined. In this paper, as in Taylor et al. (2013): the base material B shall be referred to as the background material, material A shall be referred to as the atom. The PC period, a, is consistent across the whole PC, r is the atom radius and is constant for each atom. The atom radius is expressed as a fraction of the period. All photonic crystals considered consist of a circular atom on a square lattice where the materials are either InGaP/GaAs or InGaP/air, and shall be referred to as all-semiconductor or void containing, respectively.

## Structure design

### Introduction

In this section the structure from Williams et al. (2012a) is modelled to confirm that results are similar. The structure is modified to include an additional GaAs waveguide layer (termed ballast layer) and to include an additional PC layer (termed double decker). These structures are modelled in the same way and compared to the original structure.

### Basic models

There are two main methods of calculating electromagnetic fields and their characteristics of PCSELs: finite-difference time domain (FDTD) method and models relying on coupled mode theory. FDTD method is time and computer memory consuming. Models based on coupled mode theory were initially developed for modelling of distributed feedback (DFB) lasers. These models are less demanding and in recent years they have been successfully applied to PCSELs (Sakai et al. 2006).

One of parameters coupled mode theory operates with is the coupling coefficient, K. The coefficient describes the feedback strength of a grating in DFB lasers and similarly of a PC in PCSELs. To keep the feedback high, one wants to make K high. The K provided by a PC with a square lattice has been derived and its relation with frequencies of bands has been established in (Sakai et al. 2006). In this work, we use band frequencies to estimate the coupling coefficient of the PCSEL rather than PC.

**Fig. 4:**
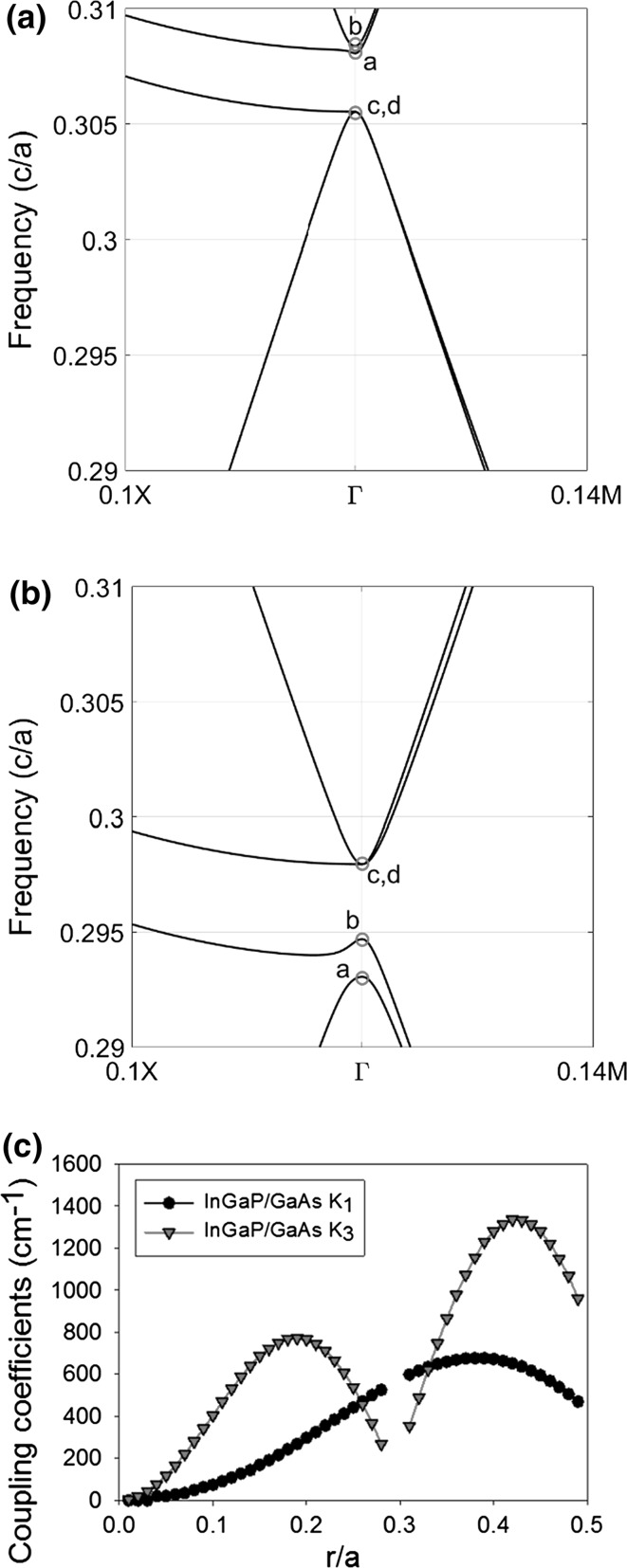
**a** Photonic band diagram showing bands a, b, c and d of all-semiconductor InGaP/GaAs PC with r/a = 0.2; **b** r/a = 0.4. Both *diagrams* calculated near Γ point. **c** Modelled coupling K_1_ (*black*) and K_3_ (*red*) for radius from 0.01a to 0.5a. (Color figure online)

This model has been shown to be successful in the past and its implementation is relatively simple. Initially the band structure for a range of r/a ratios is modelled using MIT photonic bands (MPB) (Johnson and Joannopoulos 2001). Figure 4a, b show two such band diagrams at r/a ratios of 0.2 and 0.4, respectively. Four bands, namely a, b, c, and d have been identified at Γ point in the band diagram and their normalized frequencies have been measured from these diagrams. These normalized frequencies in turn have been used to compute ω_a,b,c,d_ and the coupling coefficients analytically from Eqs. (1) and (2) (Sakai et al. 2006), where K_1_ is the in-plane coupling coefficient, K_3_ is orthogonal coupling, n_av_ is the average index of the PC and *β*_0_ = 2π/a.

1 $$\omega_{c,d} = \frac{c}{{n_{av} }}\left( {\beta_{0} + K_{3} } \right)\left( {1 - \frac{{4K_{1}^{2} }}{{\beta_{0}^{2} - K_{3}^{2} }}} \right).$$

2 $$\omega_{b} = \frac{c}{{n_{av} }}\left( {\beta_{0} - K_{3} } \right).$$

3 $$\omega_{a} = \frac{c}{{n_{av} }}\left( {\beta_{0} - K_{3} } \right)\left( {1 - \frac{{8K_{1}^{2} }}{{\beta_{0}^{2} - K_{3}^{2} }}} \right).$$

Figure 4c shows modelled coupling coefficients K_1_ (black) and K_3_ (red), for a PC consisting of InGaP/GaAs on a square lattice with a circular lattice, for a range of atom radii from 0 to 0.45 r/a. The dependence of K_3_ has two local maxima at r/a of 0.2 and 0.4, they correspond to band diagrams with largest frequency differences between bands shown in Fig. 4a, b. At around r/a = 0.3, bands cross and deduction of coupling coefficients becomes difficult.

For K_1_, the coupling coefficient generally increases as atom radius increases until a radius of 0.4, when the coupling decreases again. For K_3_, the coupling coefficient has a double peak at r = 0.15a and r = 0.4a. The coupling coefficients are not plotted at a radius of ~0.3a as this corresponds to a change in character of the band structure and the assignment bands is complicated as the bands cross (Taylor et al. 2013). The dual peak nature of K_3_ has been identified previously (Yokoyama and Noda 2005), and is similar to results shown in Taylor et al. (2013). We note that this model may be limited in its viability because it assumes that the PC is infinite. It is therefore only truly valid in the case where the electric field intensity does not vary across the thickness of the PC layer, in the case of a comparatively thick layer with a bound mode this is not necessarily true.

**Fig. 5:**
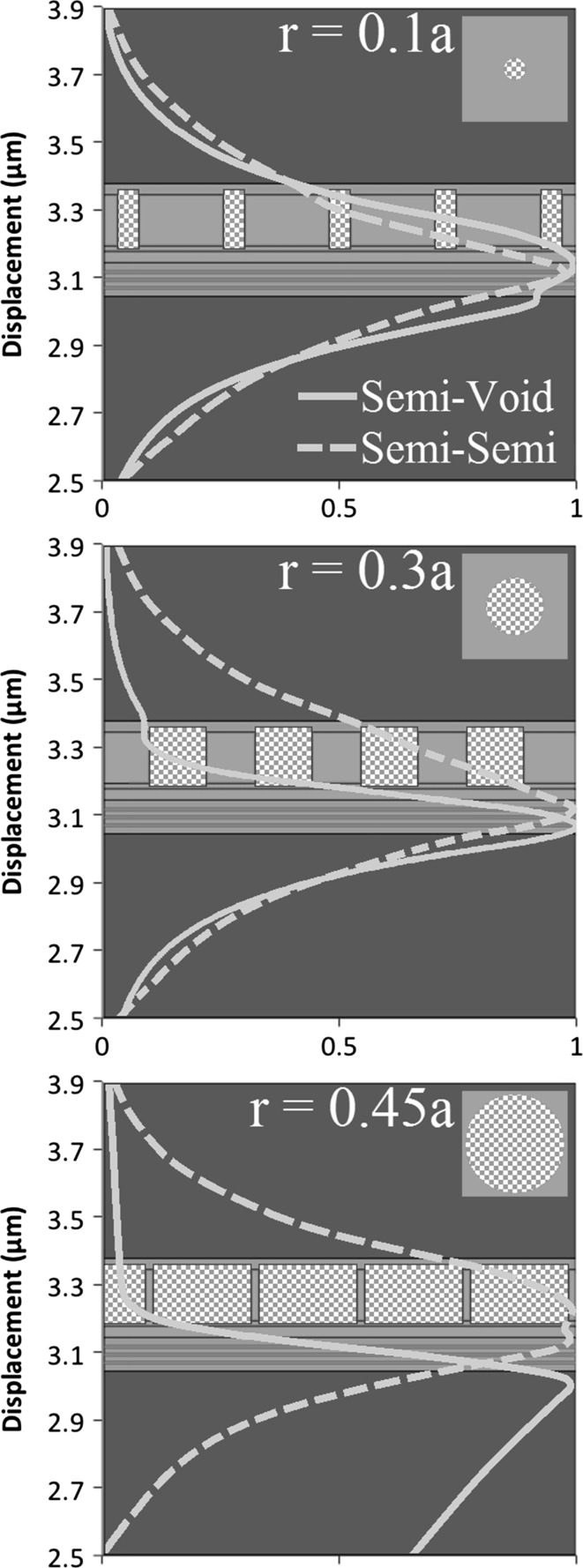
Modelled mode profile of PCSEL structure overlaid on structure schematic for atom radius of **a** 0.1a, **b** 0.3a and **c** 0.45a

Figure 5 shows the modelled mode profile of the PCSEL overlaid on a schematic of the structure described in Sect. 1, where in this case the structure is modelled as a one dimensional waveguide using FIMMWAVE (http://www.photond.com/products/fimmwave.htm). Figures a–c show the mode profile of an all-semiconductor PCSEL (dashed line) and void containing PCSEL (solid line) where the atom radius of the PC is 0.1a, 0.3a and 0.45a respectively. The mode of the void containing structure are distorted away from the PC, which is attributed to the low refractive index of the PC in the void containing case, which results in low mode overlap with the PC.

**Fig. 6:**
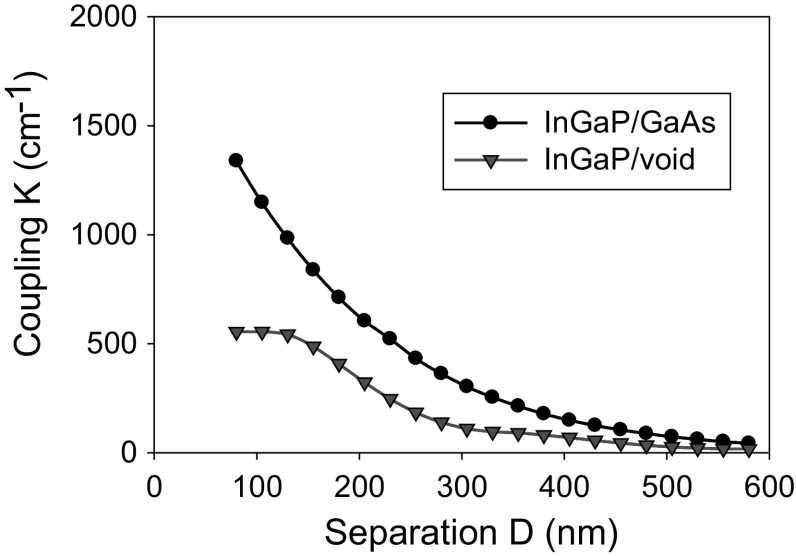
Coupling variation for separation thickness D from 40 to 600 nm for InGaP/GaAs (*black*) and InGaP/void (*red*). (Color figure online)

From the mode profile the coupling coefficient is calculated using $K = \varGamma_{PC} \frac{{2 \times\Delta n}}{\lambda }$ (where Г_PC_ is mode overlap with the PC, ∆n is the refractive index contrast and λ is vacuum wavelength). Figure 6 shows coupling as distance between active elements and PC region is increased for all-semiconductor and void containing structures. As D is increased the coupling coefficient decreases, as the PC has ever decreasing interaction with the in-plane waveguided mode. For the full range of D considered, the all-semiconductor PCSEL has a higher coupling coefficient than the void-containing counterpart. As with the previous example this model also has limitations as it considerers only the waveguide and does not consider PC effects such as feedback and scattering.

### Basic structure

**Fig. 7:**
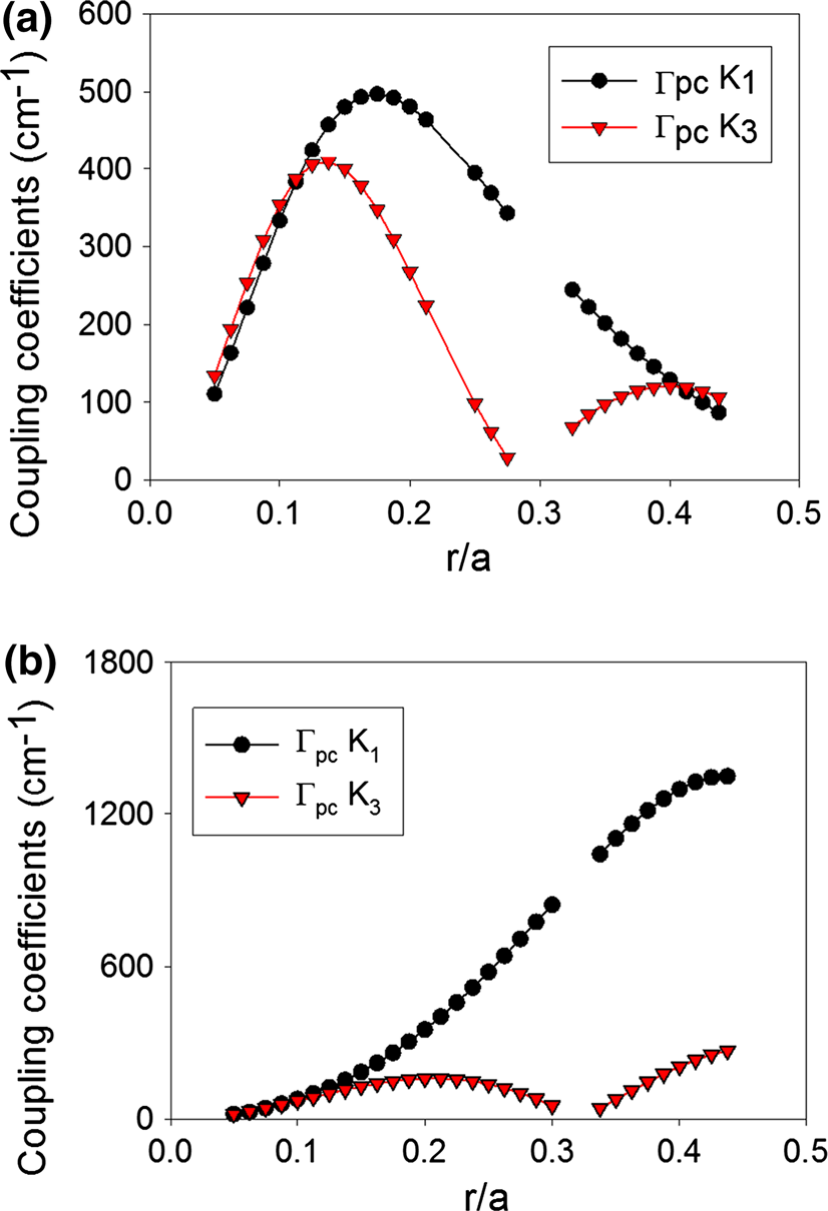
**a** Coupling coefficient K_1_ (*black*) and K_3_ (*red*) for void containing PCSEL. **b** Coupling coefficient K_1_ (*black*) and K_3_ (*red*) for all-semiconductor PCSEL. (Color figure online)

In this section we combine the two models mentioned previously to consider the effect of both the PC and the waveguide on the coupling coefficient. Here the coupling coefficient calculated for the PC from the band-structure and Eqs. (1) and (2) is multiplied by the confinement factor. This method shall be used to calculate all subsequent coupling coefficients. Figure 7 shows the coupling coefficient K_1_ (black) and K_3_ (red) for (a) void containing and (b) all-semiconductor PCSEL, for a range of atom radius from 0.05 to 0.45a. For the void containing structure, K_3_ has two peaks one at 0.15a and one at 0.4a, while K_1_ has a global peak at 0.175a. The all semiconductor PCSEL again has two peaks for K_3_, at 0.2a and 0.45a, while the K_1_ has a peak at 0.45a. The maximum peak in K_1_ is 500 cm^−1^ for the void containing structure and 1300 cm^−1^ for the all-semiconductor case. In the case of Fig. 6b mode overlap does not change significantly as r/a ratio is increased. In the case of the void containing PCSEL (Fig. 7a), when r/a is high, the low n_av_ pushes the mode away which dominates, giving a relatively low coupling coefficient. For low r/a void containing PCSELs have higher coupling coefficients, while all semiconductor PCSELs have a higher coupling at large r/a.

**Fig. 8:**
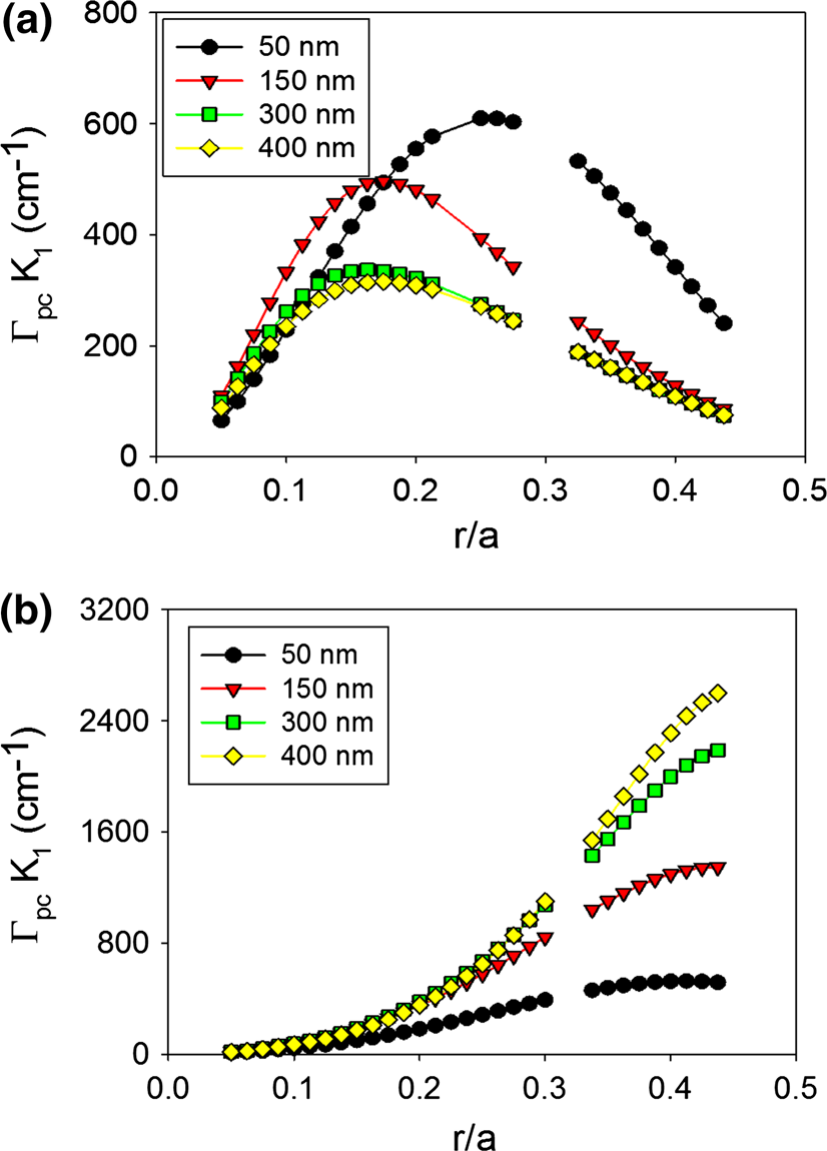
Coupling coefficient K_1_ of **a** void containing and **b** all-semiconductor PCSEL for atom radius from 0.05 to 0.45a, for PC thickness (L) of 50 nm (*black*), 150 nm (*red*), 300 nm (*green*) and 400 nm (*orange*). (Color figure online)

It is expected that the thickness of the PC layer will have an effect on the coupling coefficient of a PCSEL due to the change in modal structure and overlap integral. For the moment we ignore the need for the PC to be of specific thickness to ensure constructive interference in the far-field. Figure 8 shows the coupling coefficient K_1_ of (a) void containing and (b) all-semiconductor PCSEL where the atom radius is increased from 0.05 to 0.45a, for PC thickness (L) of 50 nm (black), 150 nm (red), 300 nm (green) and 400 nm (orange). For the void containing structure the peak coupling occurs at either 0.15a or 0.25a, and coupling decreases as PC thickness increases, this is due to the low refractive index of the PC layer further distorting the mode away from the PC layer. The all-semiconductor structure has a peak at 0.4a, increasing PC layer thickness (L) increases the coupling, this can be attributed to the PC layer having increased volume for the mode to couple to.

**Fig. 9:**
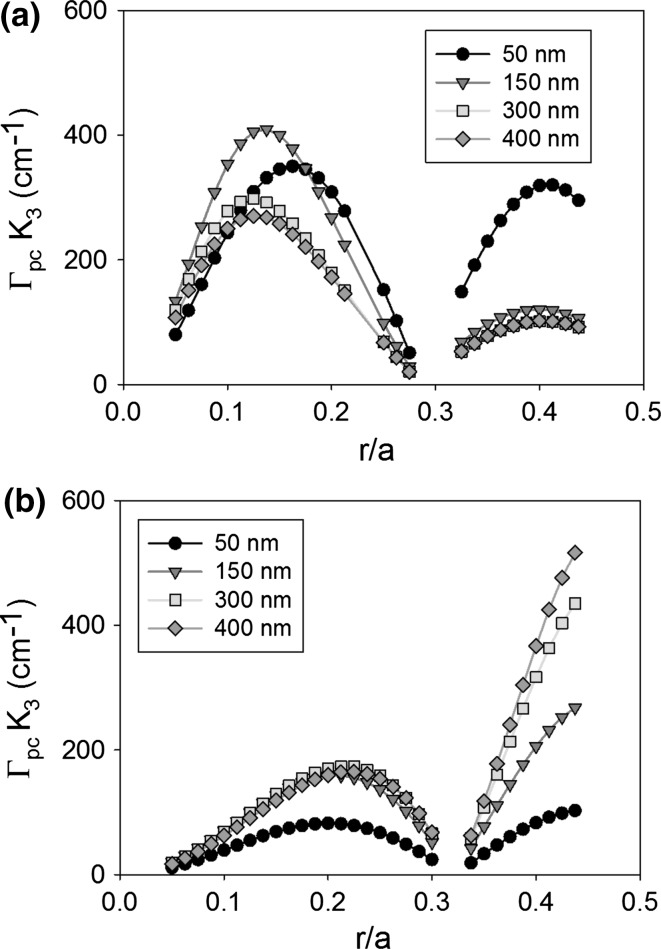
Coupling coefficient K_3_ of **a** void containing and **b** all-semiconductor PCSEL for atom radius from 0.05 to 0.45a, for PC thickness (L) of 50 nm (*black*), 150 nm (*red*), 300 nm (*green*) and 400 nm (*orange*). (Color figure online)

Figure 9 shows the same as Fig. 7 for coupling coefficient K_1_, in this case the void containing structure has two peaks at 0.125a and 0.4a, the peak coupling occurs for PC layer thickness of 150 nm. The all-semiconductor structure has a local peak in coupling at 0.2a and a global peak in coupling at 0.45a, as the PC thickness increases the coupling also increases.

In general, for this structure, we find that an increase in PC layer thickness has a monotonical improvement at an r/a of 0.4, this is enabled by the n_av_ of the PC layer being similar to the refractive index of the waveguide. In the void containing PC case, a thicker PC layer has detrimental effects due to the low n_av_. Our observed maxima in coupling at r/a = 0.2 for 150 nm PC thickness, is in agreement with observed values in the literature.

### Ballast layer structure

**Fig. 10:**
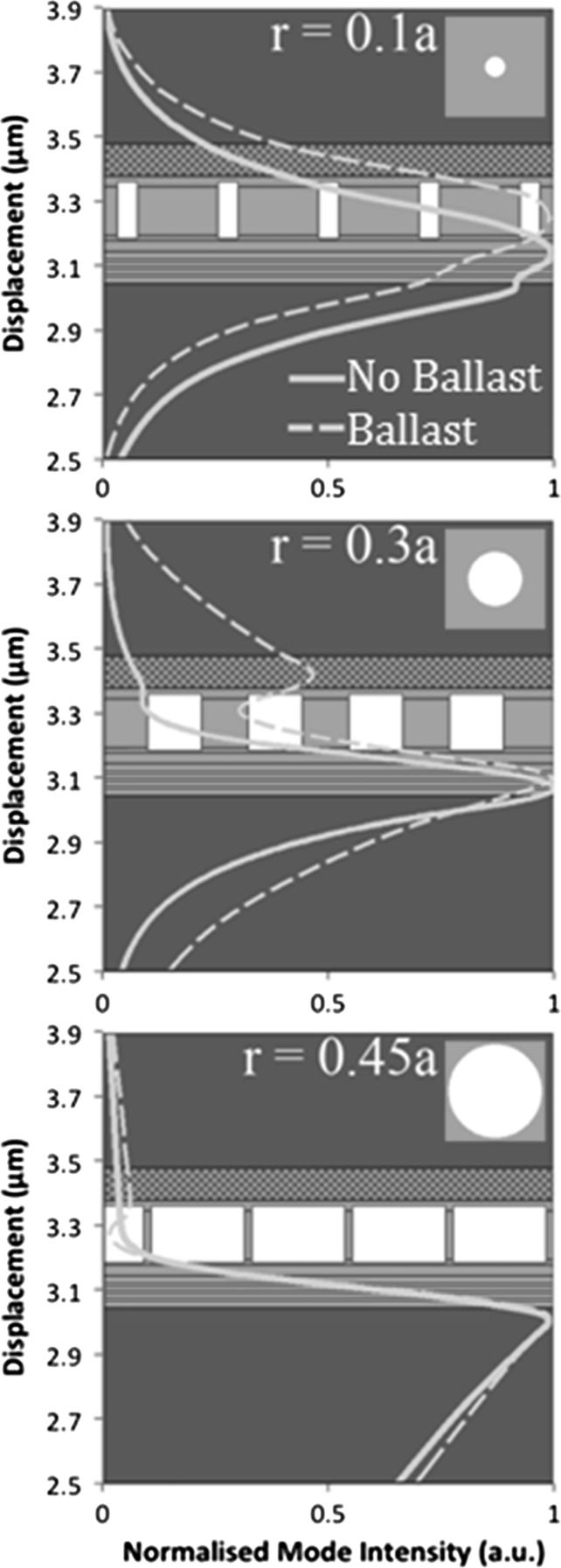
Modelled mode profile of a void containing PCSEL with a ballast layer (*dashed line*) and without a ballast layer (*solid line*), for atom radius 0.1a (**a**), 0.3a (**b**) and 0.45a (**c**)

As discussed previously, PCSEL structures containing voids have lower coupling of the optical mode to the photonic crystal as compared to their all-semiconductor counterparts. This is attributed to the low refractive index of the PC layer “pushing” the optical mode away from the PC region. In order to address this, we consider a structure which includes an additional p-type GaAs waveguide region above the PC region (of thickness T in Table 1). This additional layer is referred to as the ballast layer. This layer is intended to “pull” the mode higher in the structure and increase mode overlap with the PC region. Figure 10 illustrates this by plotting the modelled mode profile of a void containing PCSEL with a ballast layer (dashed line) and without a ballast layer (solid line), for atom radius 0.1a (a), 0.3a (b) and 0.45a (c). The mode overlap with the PC is increased for r = 0.1a and r = 0.3a. For r = 0.1a the mode peak is entirely shifted to overlap with the PC layer, this has the side effect of reducing the mode overlap with the quantum wells. At r = 0.3a a second peak in the mode profile has appeared within the ballast layer, giving an increased mode overlap with the PC layer. For r = 0.45a the inclusion of the ballast layer has little effect on the mode profile, this is due to the very low average refractive index of the PC layer in this case.

**Fig. 11:**
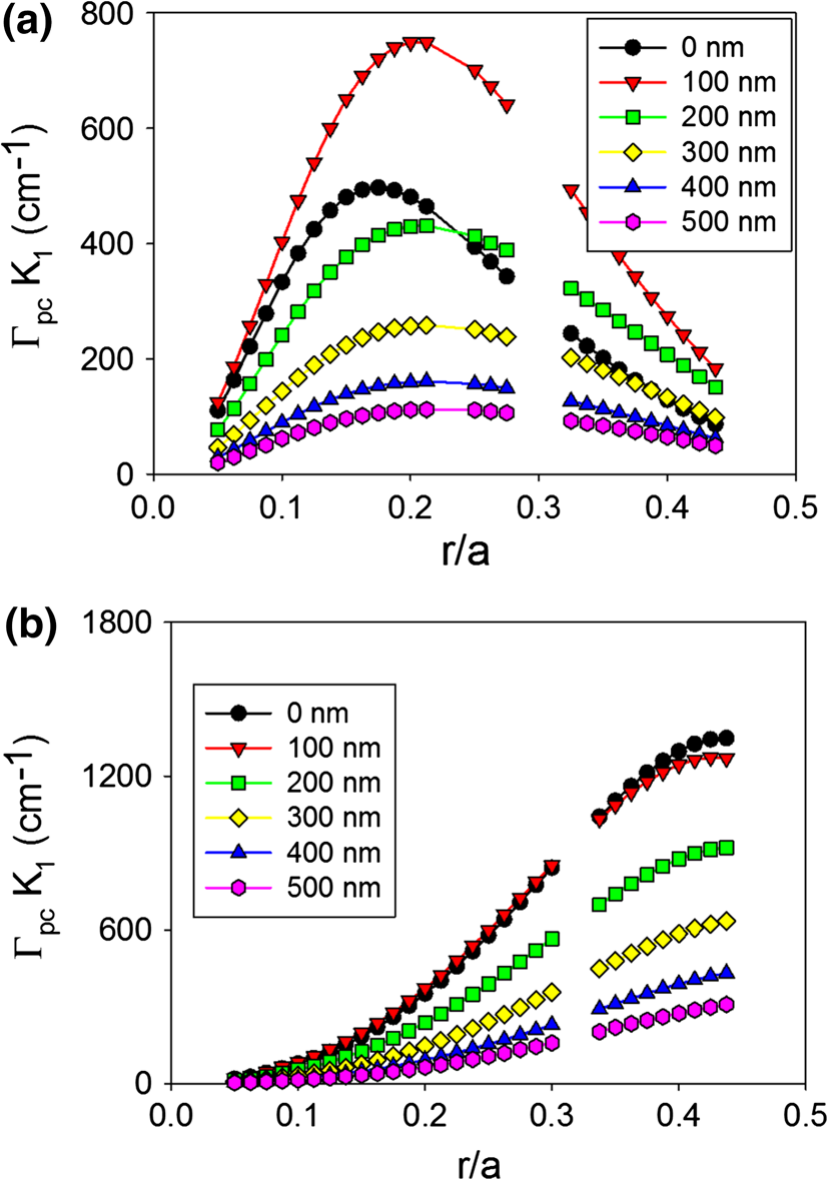
Coupling coefficient K_1_ as atom radius increases from 0.05a to 0.45a, for a void containing PCSEL (**a**) and an all-semiconductor PCSEL (**b**), for a ballast layer thickness of 0–500 nm

Figure 11 shows the coupling coefficient K_1_ as atom radius increases from 0.05a to 0.45a, for a void containing PCSEL (a) and an all-semiconductor PCSEL (b), for ballast layer thicknesses from T = 0–500 nm. For the void containing PCSEL peak coupling occurs at r = 0.2a. As ballast layer thickness (T) increases the coupling increases to a maximum of ~800 cm^−1^ at T = 100 nm, as T is increased beyond 100 nm coupling decreases. For the all-semiconductor PCSEL the ballast layer reduces the coupling coefficient, and as ballast layer thickness is increased the coupling decreases.

**Fig. 12:**
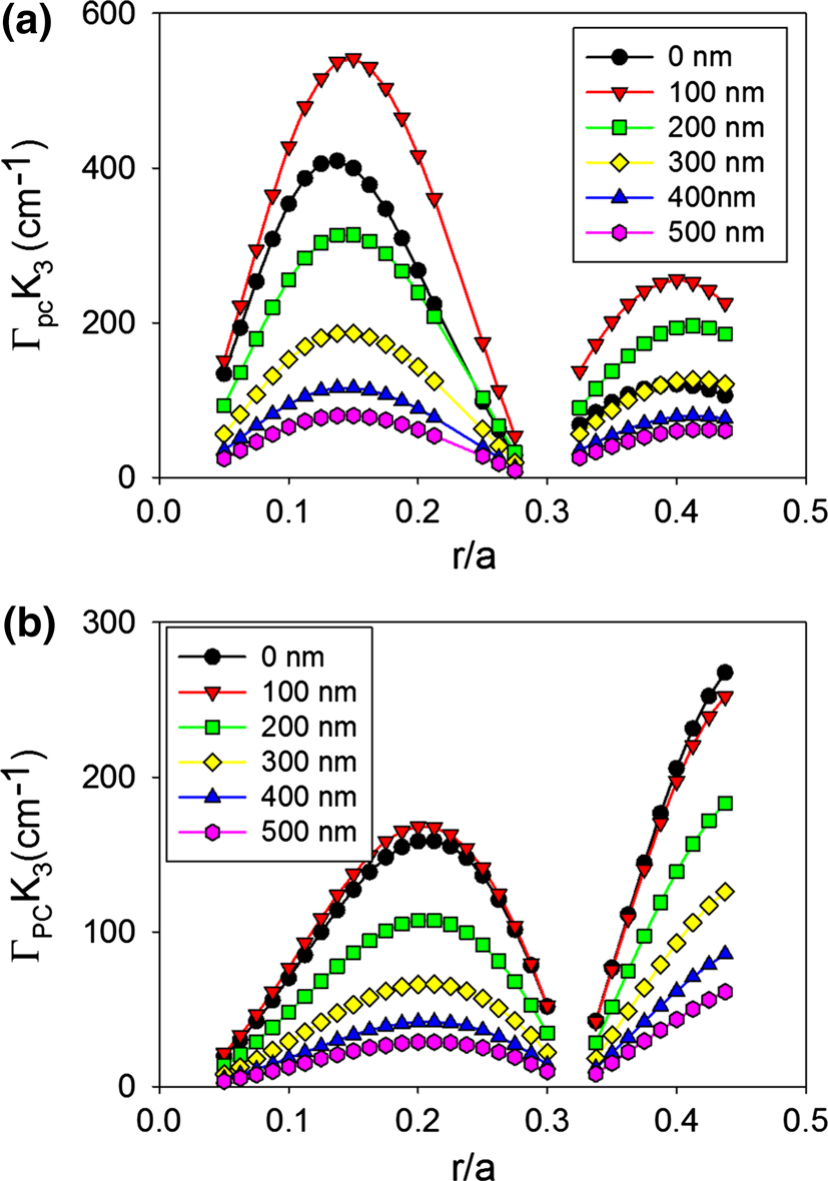
Coupling coefficient K_3_ as atom radius increases from 0.05a to 0.45a, for a void containing PCSEL (**a**) and an all-semiconductor PCSEL (**b**), for a ballast layer thickness of 0–500 nm

Figure 12 shows the same plot as Fig. 10 for coupling coefficient K_3_, in both cases a double peak is observed and coupling is increased as T is increased from 0 to 100 nm then decreases as the ballast layer thickness is increased further. For the void containing PCSEL peaks occur at r = 0.15a and r = 0.4a, while for the all-semiconductor PCSEL peaks occur at 0.2a and 0.45a.

The ballast layer has been shown to distort the mode profile of the PCSEL, for both the all-semiconductor and the void containing PCSEL a ballast layer of 100 nm gives the highest coupling. A ballast layer of suitable thickness has a significantly advantageous effect for void containing PCSELs at an r/a of 0.2. For all-semiconductor PCSEL, a marginal effect is observed with no detriment to the structural design. This bodes well for a one step epitaxial process in Williams et al. (2012a) where T may vary between runs. It is of note that the all semiconductor PCSEL has a significantly higher global maximum in coupling coefficient.

### Double decker

**Fig. 13:**
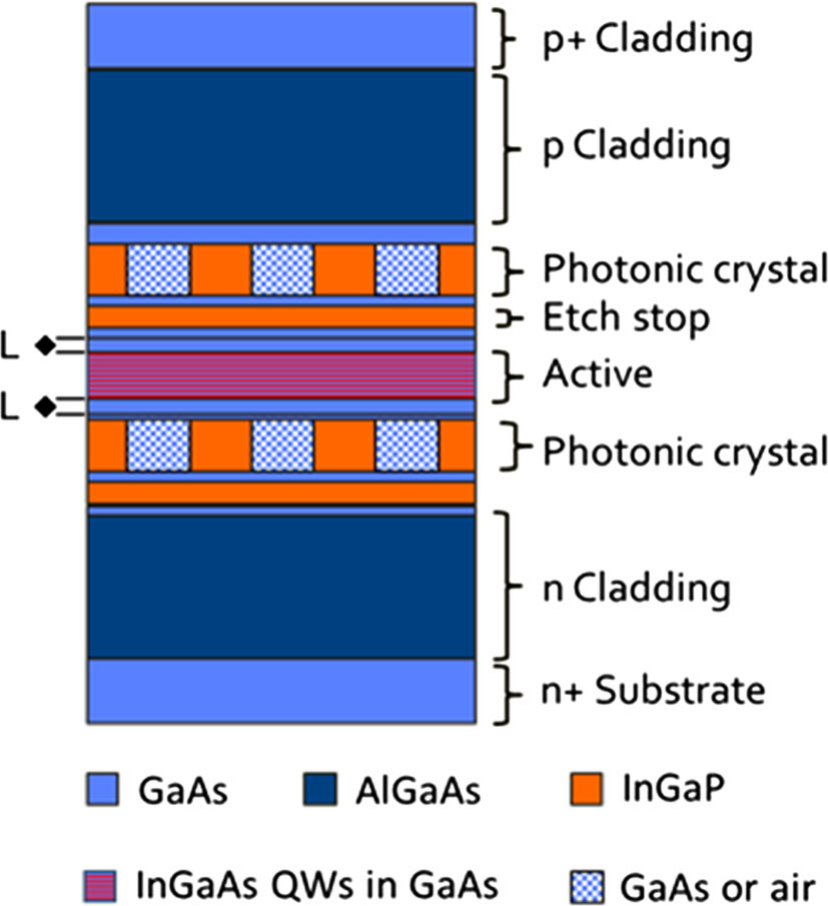
Schematic of a double decker PCSEL

The final structure considered in this section, consists of two PC regions located above and below the active region, first proposed by Kurosaka et al. (2008) The intention with this structure is that by having 2 PC regions the coupling will be increased by virtue of there being more overlap of the confined mode with the PC and the symmetry of the structure should allow the realisation of strongly bound modes for void containing PCSELs to be combined with the high index contrast that they offer. Figure 13 shows a schematic of a double decker PCSEL, where the structure consists (from bottom to top) a n-type cladding layer, an etch stop layer, photonic crystal region, a three quantum well active layer, an etch stop layer, photonic crystal region, a p-type cladding layer and finally a highly doped p-type layer. Table 2 shows the layer structure of a double decker PCSEL.

**Table 2:** Layer structure of double decker structure

Material	Thickness (nm)	Refractive index
Al_0.4_Ga_0.6_As	3000	3.31
GaAs	T	3.521
GaAs	80	3.521
In_0.48_Ga_0.52_P/GaAs or In_0.48_Ga_0.52_P/void	150	n_av_
GaAs	20 + L	3.521
In_0.48_Ga_0.52_P	40	3.143
GaAs	20	3.521
In_0.2_Ga_0.8_As	8	3.736
GaAs	20	3.521
In_0.2_Ga_0.8_As	8	3.736
GaAs	20	3.521
In_0.2_Ga_0.8_As	8	3.736
GaAs	30 + L	3.521
In_0.48_Ga_0.52_P/GaAs or In_0.48_Ga_0.52_P/void	150	n_av_
Al_0.4_Ga_0.6_As	3000	3.31

**Fig. 14:**
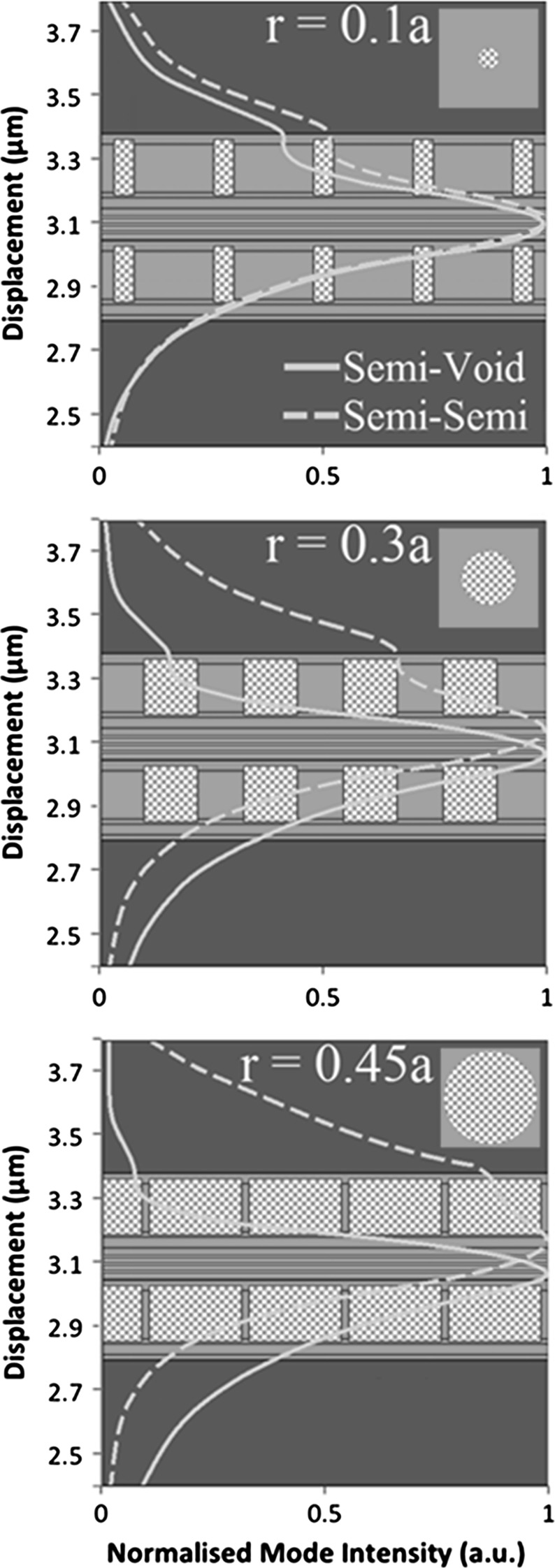
Modelled mode profile overlaid on schematic of the structure, of an all-semiconductor (*dashed*) and void (*solid*) double decker PCSEL, for atom radius 0.1a (**a**), 0.3a (**b**) and 0.45a (**c**)

Figure 14 shows the modelled mode profile overlaid on schematic of the structure, of an all-semiconductor (dashed) and void (solid) double decker PCSEL, for atom radius 0.1a (a), 0.3a (b) and 0.45a (c). In each case the mode is centred on the active region, for the all-semiconductor PCSEL, as atom radius is increased the mode is les tightly bound and mode of the mode leaks into the cladding layers. For the void containing PCSEL the mode is more tightly bound as the atom radius increases due to the symmetry of these low refractive index waveguiding layers.

**Fig. 15:**
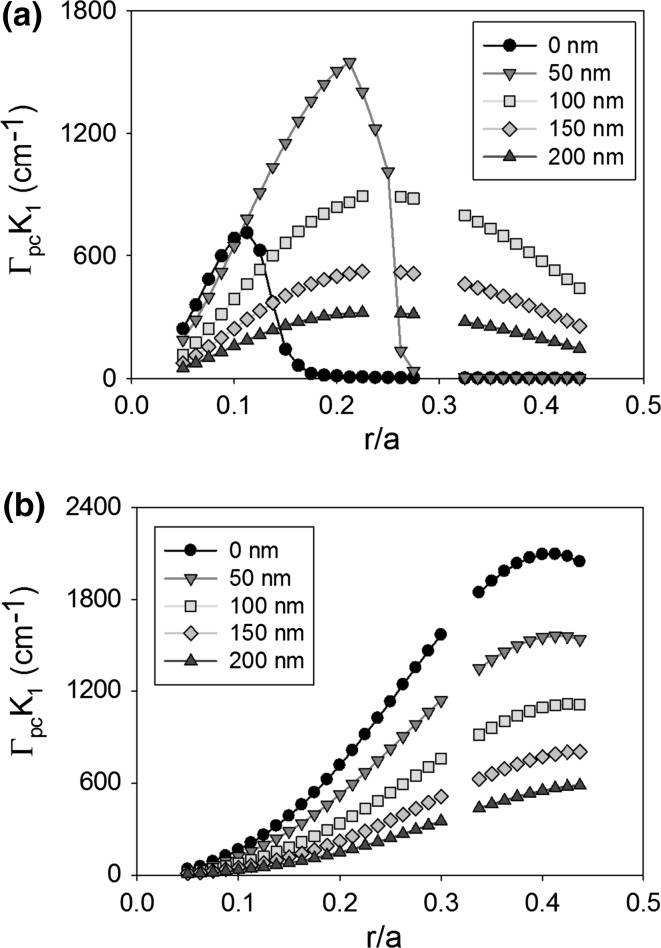
Coupling coefficient K_1_ for atom radius increases from 0.05a to 0.45a, for **a** void containing and **b** all-semiconductor double decker PCSEL, for separation distance (L) from 0 to 200 nm

Figure 15 shows the coupling coefficient K_1_ as atom radius increases from 0.05a to 0.45a, for a void containing (a) and an all-semiconductor (b) double decker PCSEL, for separation distance (L) from 0 to 200 nm. For the void containing PCSEL with separation of 0 and 50 nm the coupling is 0 above 0.2a and 0.3a, respectively, because there is no bound mode in this case, for separation distance >50 nm coupling is optimum at r = 0.25a and decreases as separation decreases. For the all-semiconductor PCSEL the coupling is greatest at r = 0.4a, as separation (L) increases the coupling decreases.

**Fig. 16:**
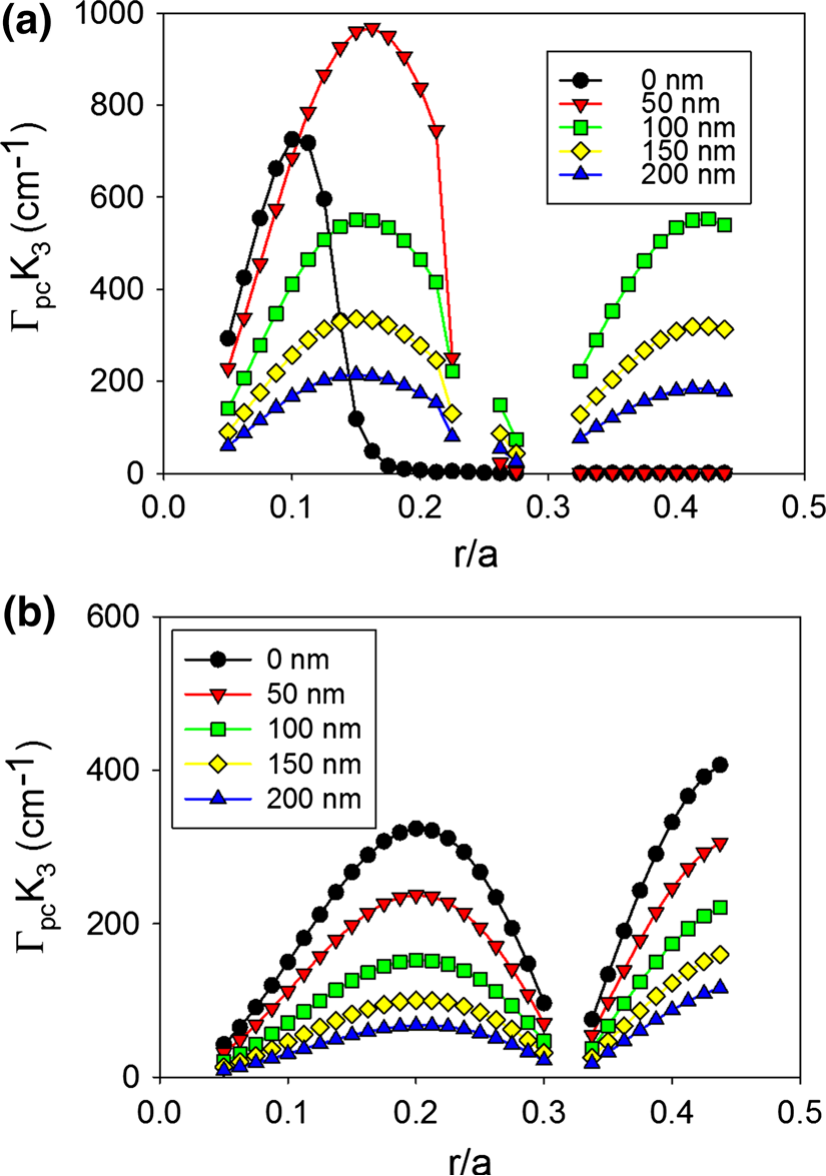
Coupling coefficient K_3_ for atom radius increase from 0.05a to 0.45a, for **a** void containing and **b** all-semiconductor double decker PCSEL, for separation distance (L) from 0 to 200 nm

Figure 16 shows the same plot as Fig. 14 for coupling coefficient K_3_, in both cases a double peak is observed and coupling is decreased as L is increased from 0 to 200 nm. For the void containing PCSEL peaks occur at r = 0.15a and r = 0.4a, while for the all-semiconductor PCSEL peaks occur at 0.2a and 0.45a. The double decker void containing PCSEL has peak coupling (K_3_) of 1000 cm^−1^ at r = 0.15a, this is a two fold increase in coupling compared with the original structure.

It is worth noting that fabricating a double decker PCSEL with wafer fusion would leave fusion interfaces within nm of the active region, which may result in defect states within the junction region of the structure. This may have significant impact on performance, reliability, and repeatability. Furthermore, the two PC layers would need to be “perfectly” aligned which would require ~nm precision over the 2–6 inch wafer which would also be challenging. Fabricating a double decker PCSEL by epitaxial regrowth would require multiple re-growths, though alignment may be less of an issue for regrown PCSELs because a feature could be etched into layer 1 to act as an alignment marker during the patterning of the PC layers. Nevertheless, “perfect” alignment would be a major technical challenge, and the double-decker PCSEL poses a number of challenges in terms of practical realisation in a robust manufacturable manner. By contrast to the previous cases, optimal void containing structures and all-semiconductor structures have similar characteristics. The key difference being the ratio of K_1_ to K_3_, the effect of comparative ratio has been discussed elsewhere (Taylor et al. 2013).

### Summary

In this Section 980 nm PCSELs based on GaAs were simulated by combining waveguide and band structure modelling. We find that n_av_ plays an important role in waveguide engineering. For an all-semiconductor PCSEL the photonic crystal has a n_av_ similar to the rest of the waveguide, allowing flexibility in waveguide design. A large r/a ratio can be achieved while a strong mode overlap (and hence high K) can be maintained. For void containing structures there is a tendency for optimal coupling to occur at radius to be at ~0.2 r/a, this is in contrast to the all-semiconductor PCSEL where optimal coupling occurs at ~0.4 r/a. The addition of a ballast layer increases the peak coupling values for both all-semiconductor and void containing PCSELs, with the largest effect for a ballast layer thickness of 100 nm. The inclusion of a second PC layer (double decker PCSEL) leads to an increase in coupling coefficients, particularly with void containing structures where the low average refractive index of the PC layer strongly confines the mode between the PC layers within the active region. However, it is worth noting that the realisation of a double decker structure poses significant fabrication issues.

## Material design

### Introduction

In this section we consider PCSEL designs for different material systems and wavelengths. The wavelengths considered are 405 nm (AlInGaN) (Kawashima et al. 2010), 1.3 µm (InGaAsP) (Noda et al. 2001) and 10 µm (AlInGaAsP QCL). Each structure is once more modelled as a 1D waveguide where the PC region is considered as a layer with a refractive index determined by an average of the PC constituents. The coupling coefficient is calculated for various separation thickness variations and for various photonic crystal thicknesses. Three PC thicknesses are considered, H, 2H and 3H where H is λ/2*n*_av_ (where λ is the vacuum wavelength and *n*_av_ is the average refractive index in the PC).

All-semiconductor PCSEL have primarily operated at 980 nm and been based on GaAs overgrowth, there has been little work on all-semiconductor PCSEL operating at different wavelengths or other materials. Previous work on a range of materials systems in the literature (Noda et al. 2001; Kawashima et al. 2010) has revolved around void/semiconductor PCSELs with comparatively small atom radius, due to the design considerations highlighted in the previous sections. However, here we wish to explore the possibilities of utilizing high mode overlap (and hence high coupling coefficient) all-semiconductor designs. As a consequence, in all cases a 50% fill factor is considered, this is taken from Sect. 2 and Taylor et al. where they find 50% fill to give a maximum in coupling. This allows simulation and comparison of PCSEL structures for a range of material systems.

### GaN/InGaN/AlGaN 405 nm structure

GaN is the material of choice for short wavelength lasers ~400 nm. This has been primarily targeted for applications in optical data storage, displays, and biomedical applications (Mitsuhashi 1997; Lefevre 2014). Incorporating a photonic crystal within a GaN laser structure would allow the aforementioned advantages of PCSELs to be realised at these wavelengths. A ~400 nm void containing PCSEL has been realised by Kawashima et al. (2010).

**Fig. 17:**
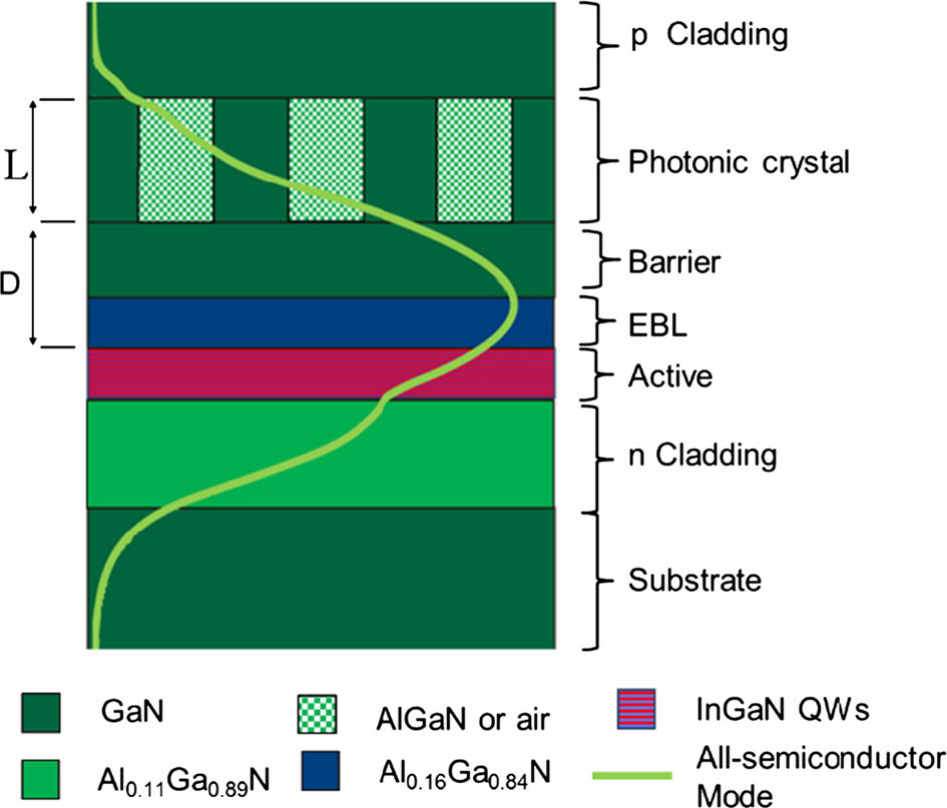
TE mode profile (intensity) overlaid on PCSEL structure for a 405 nm PCSEL

Figure 17 shows the structure of a ~400 nm PCSEL design after the layer sequence described in Kawashima et al. (2010). The structure consists of (from bottom to top) n-type Al_0.11_Ga_0.89_N cladding layer, followed by an 80-nm-thick n-doped GaN layer, multiple quantum wells (MQWs), an 80-nm-thick undoped GaN layer, a 20-nm-thick p-A_0.16_Ga_0.84_N electron-blocking layer (EBL), a 115-nm-thick p-GaN layer, a PC layer, a 40-nm-thick p-GaN layer and a 115-nm-thick p+-GaN contact layer. The MQW active zone consists of three 2.5-nm-thick In_0.09_Ga_0.91_N well layers and 7.5-nm-thick GaN barrier layers. The PC region is 220 nm thick and consists of GaN/Al_0.11_Ga_0.89_N with a 50% fill factor. The mode profile is shown overlaid on the device structure (Fig. 17). The mode profile can be seen to significantly overlap with both the photonics crystal and active regions. Refractive indices are taken from Laws et al. (2001).

**Fig. 18:**
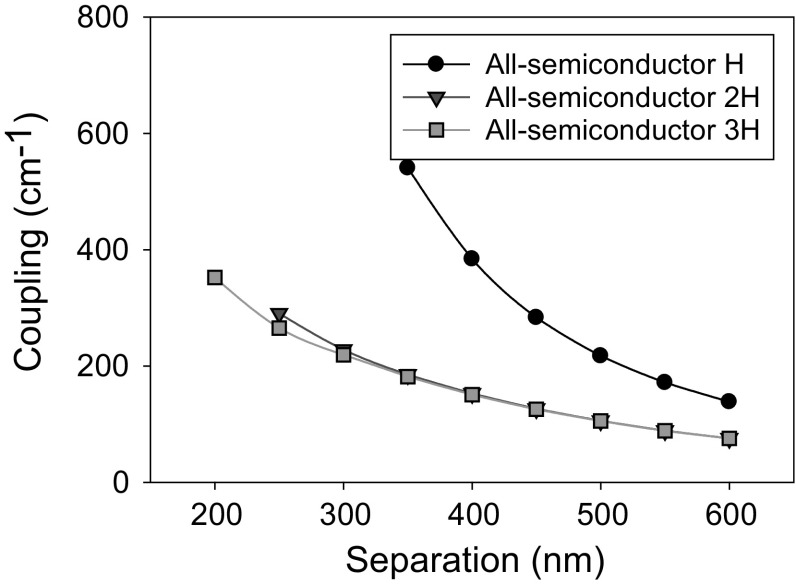
Coupling variation for separation width from 150 to 600 nm for GaN based 405 nm PCSEL

Figure 18 shows coupling as the PC separation, D, increases for GaN based 405 nm PCSEL, from Kawashima et al. (2010). The separation between the PC and the active layer, D, is varied from 150 to 600 nm and the PC thickness is H, 2H and 3H. These thicknesses are chosen to ensure constructive interference in the far-field.

**Fig. 19:**
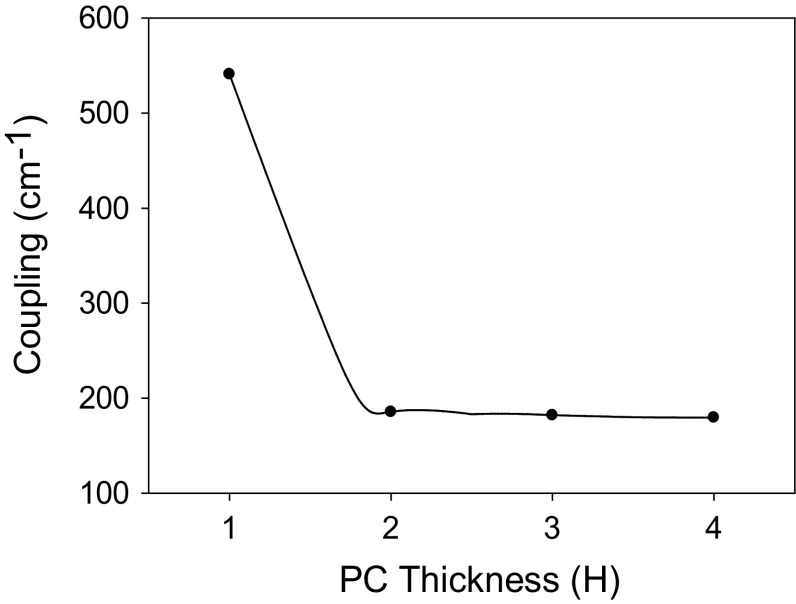
Coupling variation for 400 nm PCSELfor a variation in PC thickness H

Figure 19 shows the PC coupling of a 400 nm PCSEL shown in Fig. 17, for an all-semiconductor PC where the PC thickness is increased. The coupling decreases rapidly as PC thickness increases from 1H to 2H. As thickness increases from 2H to 4H the coupling decreases steadily.

In all cases the coupling decreases as the separation thickness D increases, and as expected the coupling tends to zero for large values of D. While increasing the thickness of the PC initially reduces the coupling, further increases in the thickness gives no change to the coupling. No values of coupling are shown for thickness of H, where Separation is <350 nm as there are no modes are bound in this case.

### InP/InGaAsP 1.3 µm structure

The absorption and dispersion characteristics of optical fibre gives two wavelength windows for optical communications at 1.55 and 1.3 µm (Mitschke 2009). Incorporating a photonic crystal within a InP laser structure would allow the advantages of PCSELs for optical communication applications. In particular, low divergence circular beams may allow lens-less optical communications modules to be considered. Furthermore, there are possible advantages in terms of manufacturing test and validation for surface emitters.

**Fig. 20:**
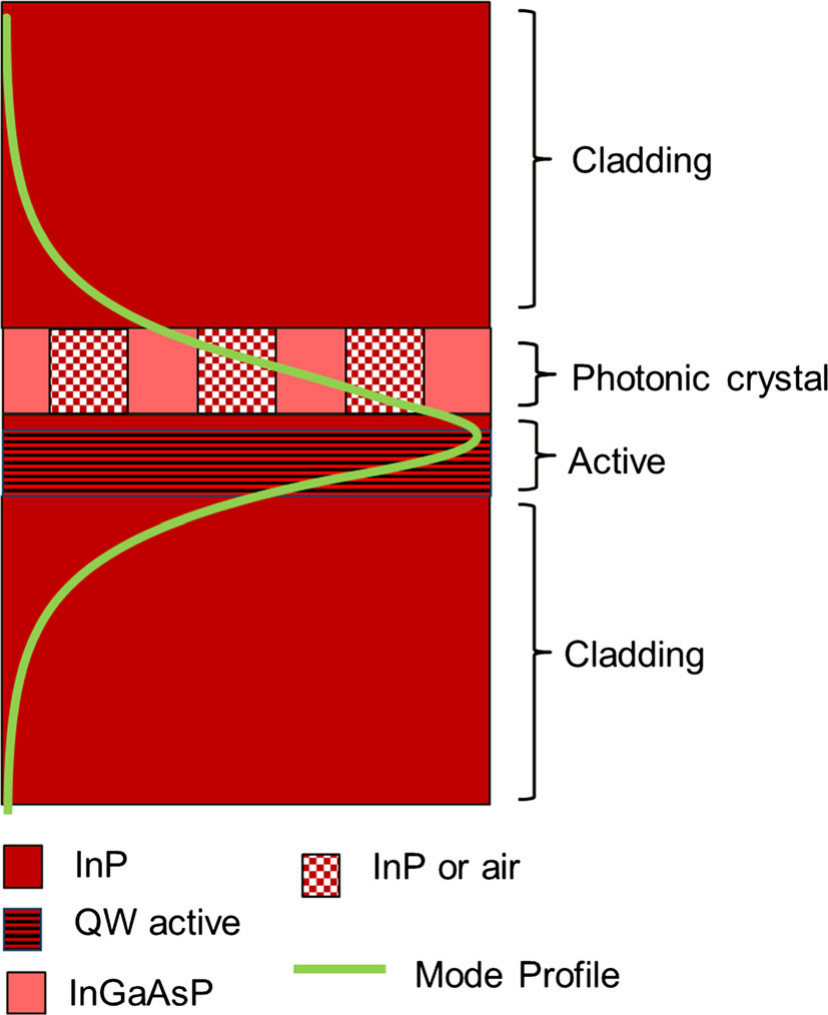
TE mode profile overlaid on PCSEL structure for an all-semiconductor 1300 nm PCSEL

Figure 20 shows the structure of a 1.3 μm PCSEL which is based on a structure from Imada et al. (1999) consisting (from bottom to top): InP substrate, 1.4 μm cladding layer, 240 nm QW active layer consisting of seven 7 nm InGaAsP quantum wells with 15 nm InP barriers, a photonic crystal consisting of InGaAsP/InP with a 50% fill factor and a 1.4μ InP cladding layer. Modelled mode profile where the mode for an all-semiconductor PC (solid line) is shown overlaid on the device structure. As observed in GaN devices the mode shows significant overlap with the PC and active layers.

**Fig. 21:**
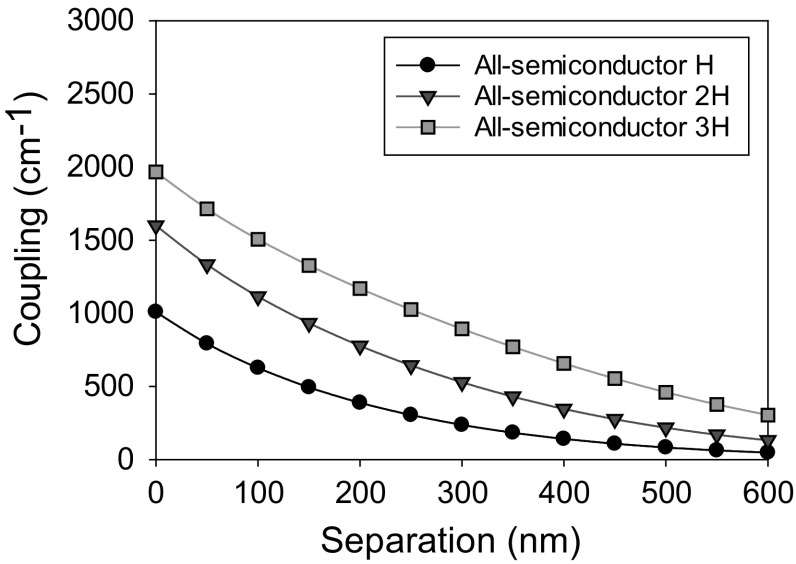
Coupling variation for separation width from 0 to 600 nm for 1.3 µm PCSEL showing all-semiconductor and void containing structures

Figure 21 shows the modelled photonic crystal coupling of a 1.3 μm PCSEL where the separation between the photonic crystal and the active region D, is varied from 0 to 600 nm where PC thickness is H, 2H and 3H. In all cases coupling approaches a maximum as separation approaches zero, as separation increases coupling decreases. Increasing the thickness of the PC layer increases the coupling, with a maximum occurring at separation of 0 nm and PC thickness of 3H, giving a maximum in coupling of 2000 cm^−1^. As expected, for large values of separation the coupling tends to zero.

**Fig. 22:**
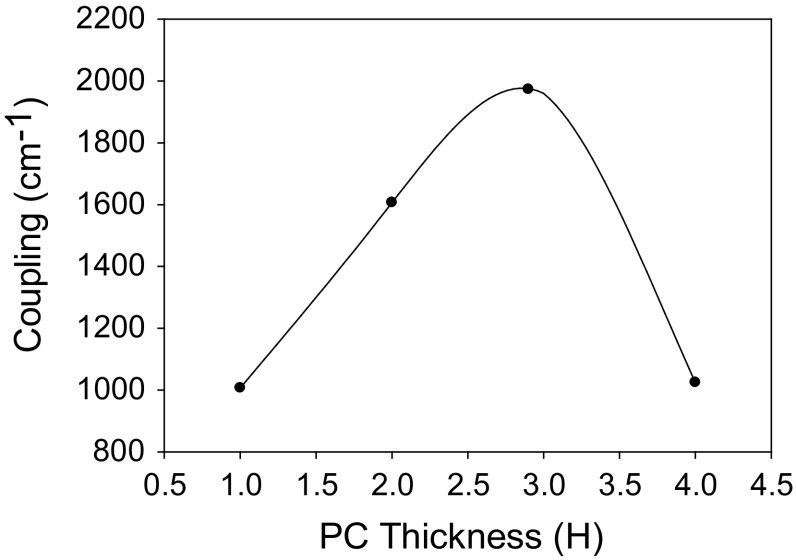
Coupling variation for all semiconductor 1.3 µm PCSEL for a variation in PC thickness H

Figure 22 shows the PC coupling of a 1.3 µm PCSEL shown in Fig. 20, where the PC thickness is increased. The coupling increases as PC thickness increases from 1H to 4H and decreases as thickness is increased further, indicating that the ideal PC thickness is 3H.

### InP/AlInAs/InGaAs 10 µm structure

InP based quantum cascade lasers (QCLs) have shown promise in a range of applications including security applications such as target illumination and industrial applications such as gas sensing (Werlea et al. 2001). Here, power scaling with area and the low divergence of the PCSEL all offer significant advantages for systems applications.

**Fig. 23:**
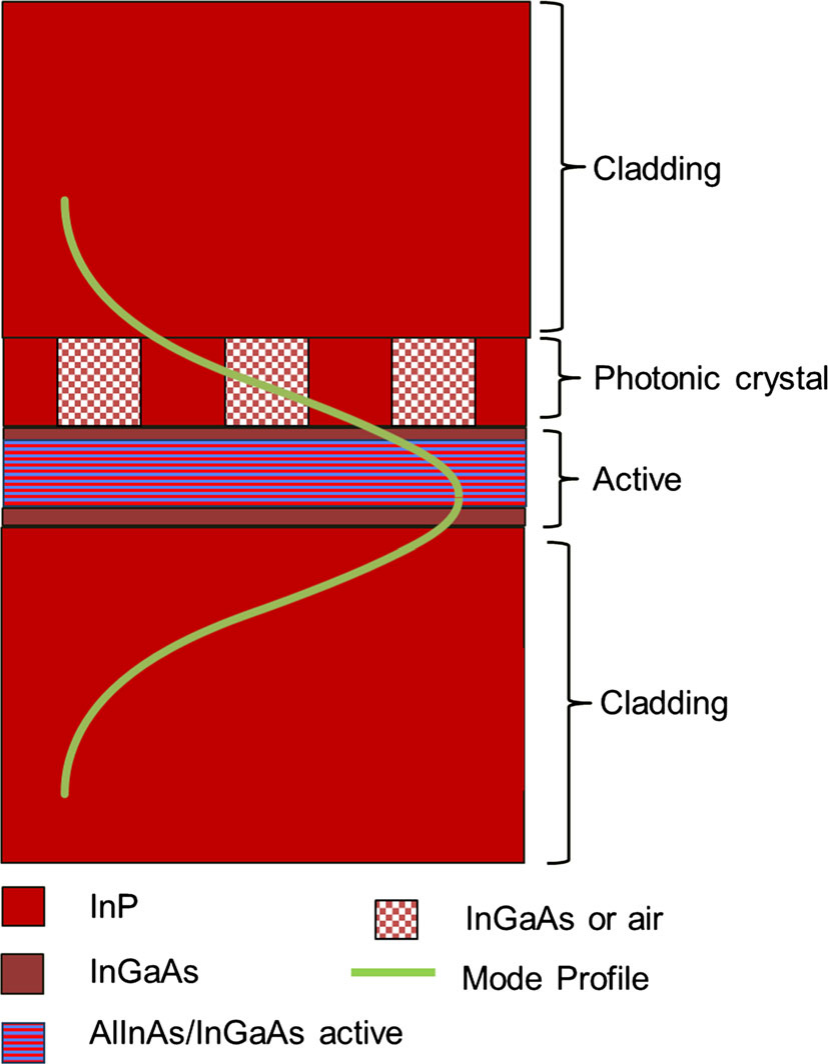
TM mode profile overlaid on InP 10 µm PCSEL structure

Figure 23 shows a 10 μm quantum cascade PCSEL based on edge emitting DFB laser work by Kennedy et al. (2007). The structure consists of (from bottom to top): 2.5 μm InP cladding layer, 270 nm InGaAs barrier layer, active region consisting of 35-stage region with the nominal layer structure of (thicknesses in Angstroms); **35**/23/**8**/66/**9**/64/**9**/58/**20**/40/**12**/40/**12/**40/**13**/39/**17**/38/**21**/35/**22**/35 where bold refers to InAlAs and normal type refers to InGaAs, a photonic crystal region which consists of InP/InGaAs and has a 50% fill factor, and a 3.5 μm InP upper cladding layer. The modelled mode profile of a 10 µm InP QCL PCSEL where the mode profile is shown overlaid on the device structure. Refractive indices are taken from Li et al. (2001). The mode considered for this structure (unlike previous structures) is TM polarised. As in previous structures the mode is shown to have a large mode overlap with both the photonic crystal and active regions.

**Fig. 24:**
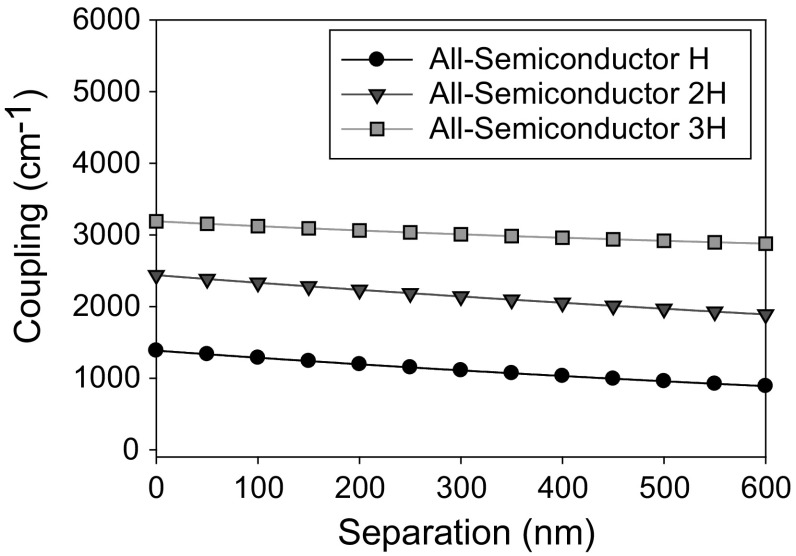
Coupling variation for separation width from 0 to 600 nm for InP based 10 µm QCL

Figure 24 shows the photonic crystal coupling of a 10 μm QCL PCSEL where the separation between the photonic crystal and the active region, D, is varied from 0 to 600 nm and the PC is all-semiconductor. The thickness of the photonic crystal layer is H, 2H and 3H. Coupling is observed to decrease steadily as separation increases with highest coupling of 3000 cm^−1^ when separation is 0 nm and thickness is 4 H.

**Fig. 25:**
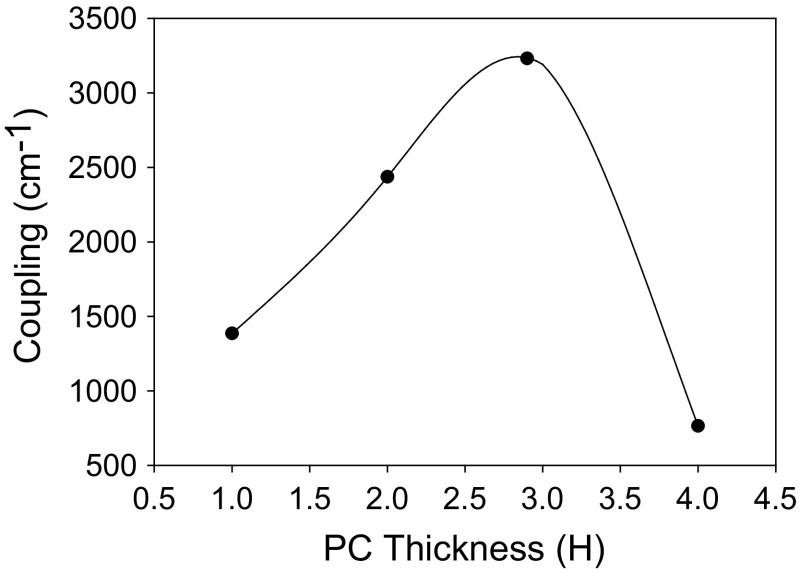
Coupling variation for InP based 10 µm QCL for a variation in PC thickness H

Figure 25 shows the PC coupling of the 10um InP QCL as a function of PC thickness. The coupling increases as PC thickness increases from 1H to 3H and decreases as thickness in increased further, indicating that an ideal PC thickness of 3H.

### Summary

In this section PCSELs operating at key wavelengths were simulated by combining waveguide and band structure modelling. We find that n_av_ plays an important role in waveguide engineering. For InP structures we find that the peak photonic crystal coupling occurs at a thickness of 3H while for GaN material systems we find that a thickness of 1H gives the highest coupling. In all cases we find that the coupling decreases as the separation of the photonic crystal and the active layer is increased. We show that in each case it is possible to obtain strong mode overlap with both the photonic crystal and the active regions. This opens a route to realise PCSELs spanning a range of wavelengths from UV to IR.

## Conclusions

In this paper we have considered three structures, a structure from Williams et al. (2012a), a ballast layer structure and a double decker structure. The coupling coefficient was calculated as a combination of the mode profile (calculated from FIMMWAVE) and the coupling calculated from the band diagram (Sakai et al. 2006), and each structure was modelled having an all-semiconductor PC and a void containing PC. In each case considered, the all-semiconductor PCSEL had higher peak coupling (both K_1_ and K_3_). The void containing structures all had peak coupling at atom radius r ~ 0.2a, while the all-semiconductor structures had peak coupling at r = 0.4a. By including a ballast layer within the structure the coupling was increased for ballast layer thickness <100 nm. The double decker structure gave the largest increase in the coupling, but fabricating such a structure would be problematic.

Three PCSEL structures with emission spanning the UV to mid-IR (400, 1300 nm and 10 µm) have been considered. To a first approximation the device design is an existing edge emitting laser structure, with a PC layer in the upper waveguide cladding. All three structures have been modelled as a 1D waveguide and PC coupling coefficients have been calculated. We show that in each case it is possible to obtain strong mode overlap with both the photonic crystal and the active regions. The realisation of PCSELs spanning a full range of key laser wavelengths will allow the many advantages of PCSELs to be implemented into a range of laser systems.
